# Progress in Anæsthetics

**Published:** 1902-01-25

**Authors:** 


### Progress in Anæsthetics.

In reference to the causation of death produced under, or as the direct result of, anæsthesia, Mitchell Banks[1] states that, in his opinion, the majority of so-called cases of sudden collapse under anæsthetics are not sudden in reality, but danger has been threatening for some time before the collapse though the anæsthetist may not have recognised the dangerous condition in time to avert it. The axiom, "plenty of air, plenty of anæsthetic," is of the utmost importance, but whatever amount of anæsthetic is over and above that which is necessary for surgical purposes is poison. The steady administration of chloroform when there is no need for it is a very grave mistake, as it puts the patient in the most critical position possible. Surgeons perhaps err in being unduly irritated when the patient moves or cries out occasionally, making the anæsthetist prefer the danger of excess to that of a safe but inconvenient possibility of movement. The want of air is another grave danger, and he would prefer the gag and tongue forceps to allow free access for it, to pulling the jaw forward, which is not so complete, and therefore less satisfactory. Hare [2] believes that in ether administration oxygen is required in excess of that in atmospheric air, and consequently deplores the use of a rubber bag in Clover's apparatus, which necessitates vitiation with carbonic acid gas and diminishing quantity of oxygen. He would administer the ether and oxygen separately, not mixing the agents for fear of chemical combination taking place between them. This objection of vitiated air is met by McGregor Young's [3] suggestion of a linen bag to replace the indiarubber one. It is made out of three strips of "holland" accurately sewn together, with a collar and tape to fix it round the clover-bag mount. He holds that it is surgically clean, and although it does not rise and fall with expiration and inspiration to any great degree, yet the patient passes under without noisy breathing, hardly any salivation and no lividity, while the abdominal heaving is lessened. The time required for the induction of anæsthesia may be slightly prolonged (7 to 12 minutes), and the initial amount of ether is greater, but in a long operation he holds that the general amount is not much above the average. His own average being 1 oz. of ether in 8 minutes, and he has used it in some hundred cases. A further advantage claimed for it is that if ether is proving insufficient, chloroform can be sprinkled upon the bag directly without changing the mask.

That the measurement of the quantity of anæsthetic used is no true gauge of the amount the patient will require to absorb is indicated by Banks [4] who speaks of the way patients differ from each other in the amounts required to produce the same result, and by Hare,[5] who indicates that there is no way of telling the proportion of the vapour lost to that inhaled. In the struggling stage of anæsthesia Banks [6] would have us allow the patient to take a few deep breaths, in a sitting posture if need be, and then to follow the face about patiently until he sinks down overcome with the anæsthetic. If he be forcibly restrained he runs great risk. He considers the left recumbent position as the best for an anæsthetic, and emphasises the necessity for recognising early any faintness which may occur, and which should be treated by rubbing the lips and nose with a rough towel.

Jellinek [7] has performed experiments upon rabbits which tend to show that strong electric currents of definite alternations and situations of the electrodes have a use as a life-saving agency. He found that these currents which killed normal rabbits, or paralysed them, beneficially stimulated those animals which were so deeply under chloroform as to be insensitive to shocks of hot or cold or pricks in the spinal cord. The electrodes were placed in the fauces and rectum. Lowering a pole of the body of a dog under chloroform and morphia, has been found by Rudolf [8] not to produce as much rise in blood-pressure as raising a pole produces lowering of that blood-pressure. Pressure upon the abdomen was not sufficient to raise the blood-pressure in the carotid, unless it was sufficient to compress the aorta. When the spinal cord is divided below where the splanchnic nerves come out, and the legs are allowed to hang down, the blood-pressure is found to fall markedly, which shows, according to Rudolf, that the vessels in the lower part of the body are largely concerned in maintaining the general blood-pressure in this "feet-down" position.

That operation shock may occur during deep chloroform anæsthesia is shown by a case of McCardie's.[9] Gas and ether was first administered for an operation on hæmorrhoids. The apparatus being faulty chloroform was given. On the patient being deeply under, the sphincter was dilated. Immediately the respiration became crowing. A.C.E. was then given on a Silk's metal inhaler and on completion of a 5 minutes' operation the pulse failed. The crutch was removed and the head lowered and shortly the respiration and pulse returned. McCardie quotes Hewitt in writing that the worst cases of surgical shock that he has seen have been in patients who have been deeply anæsthetised.

That valvular disease of the heart is not necessarily a contraindication to anæsthetics is well brought out by the analysis of cases by Finney.[10] He has collected 142 cases of persons suffering from different forms of cardiac disease, brought to operation for different troubles, minor or grave. These cases include eight of myocarditis; in one instance chloroform was not tolerated, five persons took ether badly, showing rapid, irregular, weak pulse, cyanosis, and disturbed respiration; three took ether well. There were seven cases of aortic insufficiency; four took ether well, and three successful herniotomies under cocaine; six cases of mitral stenosis did well, and included four ether, two cocaine, and one chloroform. Two cases of aortic stenosis with carcinoma mammæ, both stood a complete operation "exceptionally well." Forty-nine cases with mitral insufficiency included 38 ether administrations, nine of cocaine, and two of chloroform. Of these five took ether badly, and five not well, though they were not anxious cases. The others all did well. In seven instances of simple hypertrophy, five took ether well, one case operated upon twice, took ether badly both times, becoming cyanotic with rapid, irregular pulse and marked ether rigor occurred at the second stage. Seven out of 46 cases with functional cardiac murmurs took the anæsthetic badly; one was apparently embarrassed by elongated hypertrophied uvula, the cause of the others was not ascertained. These cases included 42 with ether, two with cocaine, and two with chloroform. There were four cases with uncertain basal systolic murmurs, of whom one took ether badly, the remaining three well. Nine cases of arrhythmia, all took ether well.

Finney compares similar cases in healthy persons under conditions as nearly alike as possible, and comes to the conclusion from these few cases that it would appear "that in myocardial affections only do anæsthetics exert any markedly bad effects. In valvular disease their influence is very slight, but yet appreciable; in functional disturbances insignificant."

It has been stated that chloroform produces rapid degeneration of the cardiac muscle, but Stengel[11] holds that this is not the case, but that it may serve to increase an already existing myocardial disease or even initiate such, and it is more than possible that it disturbs the nervous mechanism of an already diseased heart in such a way as to cause untoward symptoms. To distinguish as far as possible between the factors of the anæsthetic and shock in the causation of cardiac disturbance, he has classified the results of studying a number of trivial gynæcological operations which were unlikely in themselves to have produced shock, dividing them into those performed on the subjects of valvular or myocardial disease, and those on persons without such. He has found in the former group persons with compensated valvular trouble before operation show some slight temporary disturbance in this compensation, though it was never serious. In cases of pre-existing myocardial disease he has noticed general flagging of vitality and slow falling off in the strength of the pulse, ensuing some days later, and in two cases there were marked cardiac disturbances and sudden death.

He considers that this cardiac flagging is a large factor in the development of post-anæsthetic pneumonia, the aspiration of secretions being an excitive cause. Pulmonary embolism may also be dependent upon the state of the myocardium, the embolus originating from a vessel thrombosed on account of the weak condition of the heart. Degeneration in the myocardium is especially frequent in uterine conditions, and hence embolism of the lungs is not infrequently met with after operations for myomata of the uterus, etc. The writer believes that the first effect of etherisation is a temporary benefit in the cardiac condition, the secondary effect is after some days unfavourable: that basal pneumonia, gastro-duodenal disturbances, and especially embolism, are in reality due to a weakened state of the heart, and dependent for the exciting cause upon the anæsthetic or the shock of operation: that the untoward effects of anæsthetics are brought about by the disturbance of the nervous mechanism, or the essential muscular automaticity rather than to sudden organic changes in the myocardium.

As Mayo[12] points out conditions of myocarditis are frequently unrecognised. In health the cardiac muscular fibre has plenty of reserve power, in disease, as when compensation is required for insufficiency, it may work well so long as no great call is made upon it for extra work, but upon this call being made, as in anæsthesia, the little reserve force is soon exhausted. Myocarditis is frequently the accompaniment of valvular disease, especially lesions at the aortic opening appearing in middle life, and is often associated with arterio-sclerosis and contracted kidney. It may follow independently upon diphtheria, influenza, gonnorrhœa, and quinsey, and become chronic.

Hare[13] does not believe that the anæsthetic itself is responsible for accidents in cases with cardiac disease. He attributes this to shock of operation, having more than once seen the subjects of such disease greatly collapsed under local anæsthesia but completely revived under the use of ether, and have stood the operation well. He urges the more frequent use of atropine to prevent excessive secretion in the upper air passages and prevent the tendency to œdema of the lungs. He says it causes a disappearance of the disturbances of respiration which is little less than extraordinary in diffuse capillary bronchitis and difficult respiration from œdema.

[1] Lancet, Nov. 16. [2] Amer. Jour. Med. Sci., Aug. [3] Brit. Med. Jour., Nov. 16. [4] Lancet, Nov. 16. [5] Amer. Jour. Med. Sci., Aug. [6] Lancet, Nov. 16. [7] Brit. Med. Jour., Dec. 7. [8] Lancet, Nov. 2. [9] Med. Chron., Aug. [10] Amer. Jour. Med. Sci., Aug. [11] Amer. Jour. Med. Sci., Aug. [12] Amer. Jour. Med. Sci., Aug. [15] Amer. Jour. Med Sci., Aug.

